# Relationship between coronary artery disease and C-reactive protein levels in NSTEMI patients with renal dysfunction: a retrospective study

**DOI:** 10.1186/1471-2369-15-152

**Published:** 2014-09-17

**Authors:** Maria Udeanu, Giordano Guizzardi, Giuseppe Di Pasquale, Antonio Marchetti, Francesca Romani, Vittorio Dalmastri, Irene Capelli, Lucia Stalteri, Giuseppe Cianciolo, Paola Rucci, Gaetano La Manna

**Affiliations:** Emergency Department, Maggiore Hospital, Bologna, Italy; Cardiology Department, Maggiore Hospital, Bologna, Italy; Department of Biomedical and Neuromotor Sciences, Section of Public Health, University of Bologna, Bologna, Italy; Dialysis Unit, Maggiore Hospital, Bologna, Italy; Department of Experimental, Diagnostic and Specialty Medicine, Nephrology Dialysis and Transplantation Unit, S. Orsola Hospital, University of Bologna, Bologna, Italy

## Abstract

**Background:**

While chronic renal damage is a condition with low-grade inflammation, the potential role of inflammation in kidney disease as a marker of cardiovascular damage is of current interest. This study analyzed the relationship between renal dysfunction, chronic inflammation, and extension of coronary atherosclerosis in patients with non-ST-segment elevation myocardial infarction (NSTEMI).

**Methods:**

This retrospective study was carried out on consecutive patients presenting with NSTEMI to Maggiore Hospital’s emergency department between January 1, 2010 and December 31, 2011. Patients’ electronic charts were reviewed to gather information on patients’ history, clinical and biochemical variables, with a special focus on inflammatory markers, coronary vessel damage, and drug treatments.

**Results:**

Of the 320 individuals in the study population, 138 (43.1%) had an admission GFR <60 mL/min/1.73 m2. Kidney dysfunction was significantly associated with age (OR = 1.09, 95% CI 1.06 to 1.12), history of heart failure (OR = 2.13, 95% CI 1.08 to 4.17), and hypertension (OR = 2.31, 95% 1.12 to 4.74). C-reactive protein (CRP) and uric acid levels were significantly increased in patients with severe renal dysfunction (SRD) by bivariate and multivariate analyses, adjusted for gender, age and comorbidities at admission. The extent of coronary artery disease (CAD) was significantly higher in the SRD group (p < 0.001). Individuals with SRD were less likely to receive immediate evidence-based therapies (62.9% vs. 76.7% and 82.0% in those with intermediate and no/mild renal dysfunction, p < 0.001). Hospital stay was significantly longer in individuals with a greater extent of CAD, diabetes, and a history of heart failure, and was borderline significantly associated with renal dysfunction (p = 0.08). Older age, CAD severity, and renal function were associated with worsening GFR during hospitalization, whereas immediate evidence-based treatment was unrelated to a GFR change.

**Conclusions:**

Among individuals hospitalized for NSTEMI, those with SRD had a more extensive CAD and a higher prevalence of pre-existing cardiovascular disease. CRP was positively correlated with renal dysfunction and the number of involved coronary vessels, confirming its potential as a biomarker. Uric acid was associated with renal dysfunction but not with the number of diseased coronary vessels.

## Background

Chronic kidney disease (CKD) is a worldwide public health problem, which is becoming increasingly relevant as life expectancy of the world’s population has increased. Patients with CKD have an increased risk of end-stage renal failure and cardiovascular disease (CVD). There is a graded and independent correlation between the glomerular filtration rate (GFR) and CVD outcomes [1].

CKD is characterized by the presence of systemic, low-grade inflammation, and researchers have recently focused on the role of inflammatory markers as links between cardiovascular and kidney disease [2, 3]. The principal aim of this study was to analyze the relationship between renal function at admission, coronary artery disease (CAD) severity, and inflammation markers in patients hospitalized for non-ST-segment elevation myocardial infarction (NSTEMI). The secondary aims were to evaluate the in-hospital outcomes and 1-year and 2-year mortality rates of these patients.

## Methods

### Study population

The study included consecutive patients presenting to the Emergency Department of Maggiore Hospital from January 1, 2010 to December 31, 2011, with a NSTEMI. NSTEMI diagnosis was based on electrocardiographic (ECG) ST-segment depression or prominent T-wave inversion and/or positive biomarkers of necrosis (e.g., troponin) in the absence of ST-segment elevation and in an appropriate clinical setting (chest discomfort or anginal equivalent) as defined in American College of Cardiology/American Heart Association guidelines [4]. The diagnostic cut-off for myocardial infarction was defined as a cardiac troponin measurement exceeding the 99th percentile of a normal reference population (upper reference limit) using an assay with an imprecision (coefficient of variation) of ≤ 10% at the upper reference limit. Our Laboratory uses the SIEMENS Dimension EXL TM integrated chemistry system. The reference interval for Troponin I was 0.000-0.056 ng/mL. [5, 6].” According to the ACC/AHA and ESC guidelines for NSTEMI management, an early invasive strategy (diagnostic angiography with intent to perform revascularization) is indicated in initially stabilized NSTEMI patients (without serious comorbidities or contraindications to such procedures) who have an elevated risk for clinical events. For patients not at high risk, a delayed invasive approach is also reasonable. The study was approved by the ethics committee of the S.Orsola University Hospital of Bologna. Data were extracted from the patients’ electronic medical records and all patient-identifying information was removed before analysis, in compliance with the Italian privacy law (Act No. 67, December 31, 1996). Patients with severe infections (sepsis, lung infections, and urinary sepsis) were excluded. No lower limit for the value of GFR was used as an exclusion criterion.

The patients were classified into three groups: no/mild renal dysfunction (≥60 mL/min/1.73 m2 intermediate renal dysfunction (31–59 mL/min/1.73 m2), and severe renal dysfunction (<30 mL/min/1.73 m2) according to their renal function at admission (aGFR). aGFR was estimated using the Chronic Kidney Disease Epidemiology Collaboration (CKD-EPI) formula, to characterize serum creatinine levels in relation to age and gender.

CAD was assessed in those patients who underwent coronary angiography. CAD severity was defined as the number of vessels with ≥1 obstructive atherosclerotic lesions, defined as lesions producing ≥50% reduction in diameter of the left main coronary artery or ≥70% reduction in diameter of the major epicardial vessels. Immediate evidence-based treatment was defined as the use of aspirin, clopidogrel, low-molecular-weight-heparin (LMWH), beta-blockers, angiotensin converting enzyme (ACE) inhibitors, and statins. Mortality rates at 1 and 2 years after discharge were obtained by linking patient data with the Sistema Informativo Politiche per la Salute e Politiche Sociali (SISEPS) database of Emilia-Romagna region.

### Statistical analysis

Continuous variables were compared using analysis of variance (ANOVA) F, Kruskal-Wallis, or median tests, followed by post-hoc pairwise tests. Categorical variables were compared using the χ^2^ test. Bonferroni correction was applied to the probability level to control for possible type-I error. Multiple logistic regression was used to predict aGFR as a function of gender, age and comorbidities at admission. Multiple linear regression analysis was used to predict the number of vessels with CAD, C-reactive protein (CRP) levels, and troponin levels as a function of aGFR, gender, age, and comorbidities at admission. The change in GFR from admission to discharge was analyzed as a function of renal function, comorbidities, severity of CAD, age, gender, and left ventricular dysfunction (defined as ejection fraction [EF] ≤40%) at admission, using multiple linear regression.

The GFR change was determined by subtracting aGFR from the discharge GFR. Therefore, a positive change indicates improvement from baseline and a negative change indicates worsening. Patients undergoing dialysis were excluded from the GFR change analysis.

Multiple linear regression analysis was also used to evaluate the relationship between the length of hospital stay and renal function, comorbidities, CAD severity, age, gender, and left ventricular dysfunction at admission. Length of hospital stay was normalized by square root transformation. All analyses were performed using SPSS, version 20.0.

## Results

### Patient admission characteristics

During the study period, 334 patients presented with NSTEMI. Fourteen were excluded from analysis: one for generalized sepsis, nine for lung infections, and four for urinary sepsis. The study population included 320 patients: 182 had no/mild, 103 had intermediate, and 35 had severe renal dysfunction. Among these 35 patients, 10 were CKD stage 5D patients on chronic hemodialysis treatment .Compared with patients with no/mild dysfunction, those with intermediate or severe dysfunction were significantly older and more likely to be female and have left ventricular dysfunction and a history of heart failure, diabetes, hypertension, peripheral artery disease, or previous MI (Table 1).

**Table 1:** **Baseline and in-hospital medication characteristics of the study sample by level of kidney function**

Variables	Group A:	Group B:	Group C:	P-value	Post-hoc
	GFR ≥60	GFR 59-30	GFR <30		Significant
	(n = 182)	(n = 103)	(n = 35)		Comparisons*
Age, mean (SD) (years)	68.7 (12.5)	81.2 (8.8)	79.5 (11.0)	<.0001	A < B,C
Female, n (%)	49 (26.9)	46 (44.7)	23 (65.7)	<.0001	A < B < C
History of lipid disorder, n (%)	106 (58.2)	59 (57.3)	15 (42.9)	0.236	-
History of diabetes, n (%)	45 (24.9)	37 (35.9)	18 (51.4)	0.004	A < B,C
History of hypertension, n (%)	130 (71.8)	87 (84.5)	29 (82.9)	0.035	A < B
History of PAD, n (%)	11 (6.1)	14 (13.6)	7 (20.0)	0.015	A < B,C
Prior myocardial infarction, n (%)	68 (37.6)	49 (48.0)	24 (70.6)	0.001	A,B < C
Prior stroke or TIA, n (%)	16 (8.8)	17 (16.5)	7 (20.0)	0.061	-
Left ventricular dysfunction (EF ≤ 40), n (%)	26 (14.3)	27 (26.2)	11 (31.4)	0.011	A < B,C
History of heart failure, n (%)	24 (13.2)	38 (36.9)	14 (40.0)	<.0001	A < B,C
*Renal function characteristics*					
GFR at admission, mean (SD) (mL/min/1.73 m^2^ )	81.1 (13.6)	45.5 (8.8)	18.1 (7.7)	<.0001	A > B > C
GFR at peak, mean (SD) (mL/min/1.73 m^2^ )	73.7 (16.4)	41.8 (10.1)	16.0 (9.3)	<.0001	A > B > C
GFR at discharge, mean (SD) (mL/min/1.73 m^2^ )	80.9 (15.8)	51.4 (15.4)	22.9 (16.9)	<.0001	A > B > C
*In-hospital medications, n (%)*					
Aspirin	180 (98.9)	99 (96.1)	27 (77.1)	<.0001	A,B > C
Clopidogrel	170 (93.4)	97 (94.2)	31 (88.6)	0.513	-
LMWH	179 (98.4)	95 (92.2)	33 (94.3)	0.037	A > B
Beta-blockers	177 (97.3)	93 (90.3)	32 (91.4)	0.036	A > B
ACE inhibitors	163 (91.1)	91 (88.3)	22 (62.9)	<.0001	A,B > C
Statins	152 (84.4)	79 (77.5)	19 (54.3)	<.0001	A,B > C
Immediate evidence-based treatment, n (%)	162 (89.0)	79 (76.7)	22 (62.9)	<.0001	A > B,C

*Bonferroni-corrected p value was used for Post-hoc analysis.

*Abbreviations:* *GFR* glomerular filtration rate, *SD* standard deviation, *PAD* peripheral arterial disease, *TIA* transient ischemic attack, *EF* ejection fraction; *LMWH* low-molecular-weight-heparin, *ACE* angiotensin-converting enzyme.

Patients with mild and moderate renal dysfunction had a significantly higher diastolic arterial pressure (DAP) and mean arterial pressure (MAP) than patients with severe renal dysfunction (DAP: 80 ± 12 mm Hg and 79 ± 14 mm Hg vs. 72 ± 12 mm Hg , respectively; ANOVA F = 6.3, p = 0.002; MAP: 100 ± 17 mm Hg and 100 ± 14 mm Hg vs. 92 ± 17 mm Hg, respectively, ANOVA F = 4.1, p = 0.018). Systolic blood pressure did not differ significantly among the three aGFR groups (ANOVA F = 1.781, p = 0.0170).

During multiple logistic regression including age, gender, and comorbidities at admission, kidney dysfunction was significantly associated with age (odds ratio [OR] = 1.09, 95% confidence interval [CI] = 1.06 to 1.12), heart failure history (OR = 2.13, 95% CI = 1.08 to 4.17), and hypertension (OR = 2.31, 95% CI = 1.12 to 4.74).

Regarding evidence-based drug treatment, patients with severe renal dysfunction were less likely to receive ACE inhibitors, statins, and aspirin than patients with no/mild dysfunction; those with intermediate renal dysfunction were less likely to receive beta-blockers and LMWH (Table 1).

Overall, a majority of patients with no/mild and intermediate renal dysfunction received immediate evidence-based treatment, but only 62.9% of those with severe renal dysfunction received this treatment.

### Coronary artery disease severity, inflammatory markers, and renal dysfunction

Three-vessel lesions were more common in patients with severe (83.3%) and intermediate (50%) renal dysfunction than in patients with no/mild dysfunction (32.3%) (Table 2).

**Table 2:** **Relationship between myocardial damage, inflammation, and severity of coronary artery disease by level of kidney function**

Variables	Group A:	Group B:	Group C:	P value	Post-hoc
	GFR ≥60	GFR 59-30	GFR <30		Significant
	(n = 182)	(n = 103)	(n = 35)		Comparisons*
*In hospital markers*					
Troponin I, median (IQR), ng/mL	1.2 (0.33-6.46)	2.3 (0.48-6.51)	2.9 (0.61-6.27)	0.052	A < C
CRP, median (IQR), mg/mL	0.3 (0.17-1-13)	0.69 (0.23-2.65)	1.9 (0.73-7.67)	<0.001	A,B < C
Uric acid, median (IQR), mg/mL	5.9 (4.95-7.2)	7.5 (6.1-9.4)	7.2 (5.8-8.3)	<0.001	A < B,C
*Angiographic findings, n (%)*†				0.002	
No CAD	14/167 (8.3)	4/70 (5.7)	0		-
1- and 2-vessel CAD	99/167 (59.3)	31/70 (44.3)	2/12 (16.7)		A > C
3-vessel CAD	54/167 (32.3)	35/70 (50)	10/12 (83.3)		A < B,C

*Bonferroni-corrected p value was used for Post-hoc analysis.

*Abbreviations:* *GFR* glomerular filtration rate, *IQR* interquartile range, *CRP* cross-reactive protein, *CAD* coronary artery disease.

† In the 249 patients who underwent coronary angiography.

CRP and uric acid (UA) were significantly higher in patients with severe renal dysfunction (Table 2). Distinguishing ESRD patients on dialysis treatment and patients with GFR < 30 ml/min/1.73m2 on conservative therapy, we compared the groups in terms of inflammatory markers: differences previously identified remained significant (CRP median [IQR]: 1.3 (0.48-5.50) vs 2.9 (1.14-10.89). When the relationships between inflammatory markers and renal dysfunction (as a continuous variable) were analyzed using multiple linear regression and adjusted for comorbidities, age, and gender, CRP and UA remained significantly associated with the degree of renal dysfunction.

**Figure 1:**
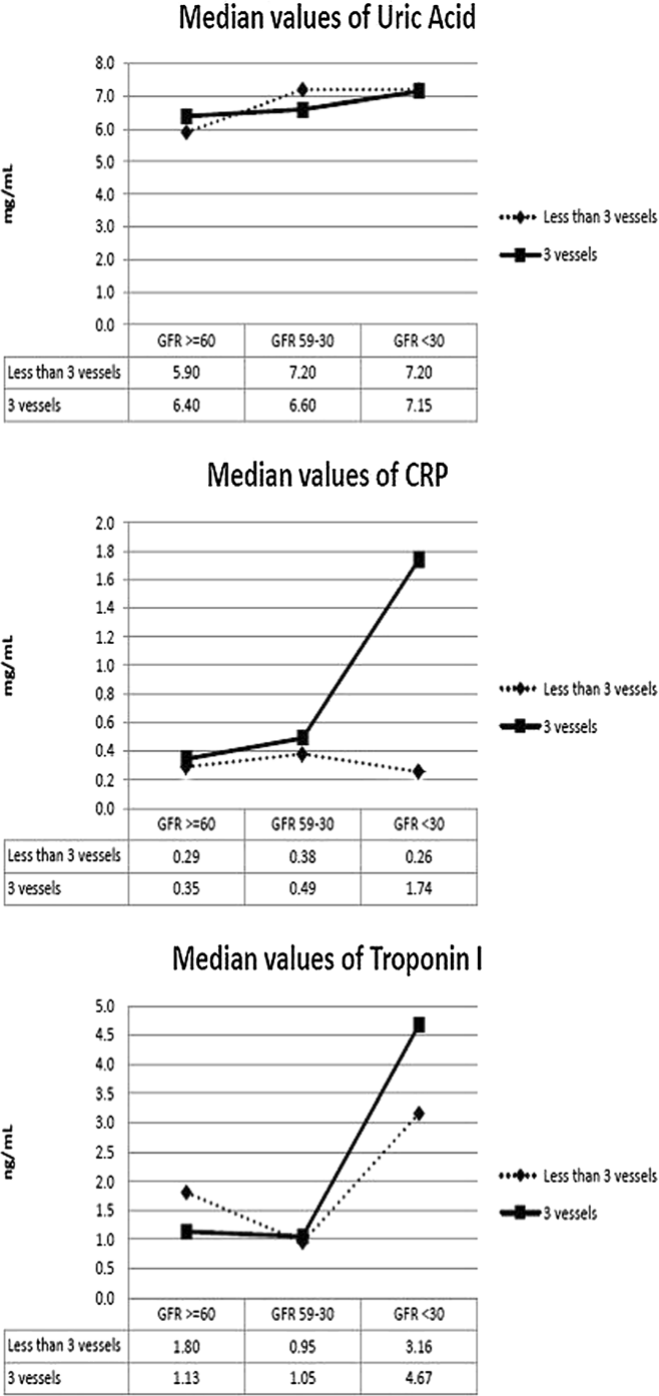
**Median levels of inflammatory markers as a function of renal dysfunction and number of coronary vessels involved.** Abbreviations: CRP, C-reactive protein.

Figure 1 shows the relationship between troponin I, CRP, UA, renal dysfunction severity, and number of vessels with lesions in the 249 patients who underwent coronary angiography. Only CRP was significantly associated with CKD severity in patients with three-vessel CAD. CRP levels were significantly higher in patients with three-vessel lesions than in those without three-vessel lesions (CRP mean ± standard deviation [SD]: 1.07 ± 2.26 vs. 1.79 ± 2.75, p = 0.03). UA levels were significantly higher in patients with severe and intermediate renal dysfunction compared to those with no/mild renal dysfunction (Table 2), but were unrelated to the number of vessels involved (UA mean ± SD (3vessels CAD vs. <3vessels CAD): 6.46 ± 2.07 vs. 6.79 ± 1.81, p = 0.204).

### In-hospital renal and other outcomes

The length of hospital stay increased linearly with the level of renal dysfunction, from a mean of 5.6 days to 8.2 days (Table 3). During multiple linear regression analysis of the 249 patients with angiography results, the length of hospital stay was significantly associated with the extent of CAD, presence of diabetes, and history of heart failure, and borderline significantly associated with renal dysfunction (p = 0.08) (Table 4).

**Table 3:** **In-hospital outcomes**

Variables	Group A:	Group B:	Group C:	P value	Post-hoc
	GFR ≥60	GFR 59-30	GFR <30		Significant
	(n = 182)	(n = 103)	(n = 35)		Comparisons*
*In hospital outcomes*					-
Length of hospital stay, mean (SD) (days)	5.6 (4.9)	7.9 (6.3)	8.2 (4.8)	0.001	A < B < C
In-hospital death, n (%)	3 (1.6)	1 (1.0)	2 (5.7)	0.193	-

*Bonferroni-corrected p value was used for Post-hoc analysis.

*Abbreviations:* *SD* standard deviation.

**Table 4:** **Predictors of length of hospital stay: multiple linear regression analysis results**

Independent variables	b	P value	95% Confidence interval
Gender (male)	0.02	0.87	-0.173 to 0.204
Age (years)	0.00	0.74	-0.007 to 0.009
Immediate evidence-based treatment	-0.19	0.18	-0.464 to -0.090
GFR at admission (mL/min/1.73 m^2^ )	0.00	0.08	-0.008 to -0.001
Number of vessels with CAD	0.12	**0.01**	0.029 to 0.217
Left ventricular dysfunction (EF ≤40)	0.11	0.38	-0.133 to 0.349
History of heart failure	0.45	**<0.001**	0.211 to 0.681
History of PAD	0.12	0.44	-0.184 to 0.420
History of diabetes	0.26	**0.01**	0.072 to 0.446
History of lipid disorders	-0.13	0.13	-0.307 to 0.040
History of hypertension	0.00	0.99	-0.196 to 0.194
Prior myocardial infarction	-0.12	0.20	-0.299 to 0.064
Prior stroke or TIA	0.16	0.27	-0.123 to 0.445

*Abbreviations:* *GFR* glomerular filtration rate, *CAD* coronary artery disease, *EF* ejection fraction, *PAD* peripheral arterial disease, *TIA* transient ischemic attack.

Values in bold are statistically significant.

The mean GFR of all patients (excluding the 10 receiving dialysis) improved from admission to discharge by 2.3 mL/min/1.73 m2 (SD = 12.5). The percentage change in GFR was 0.4%, 13.2%, and 32.3% in patients with no/mild, moderate, and severe admission renal dysfunction, respectively. Older age, CAD severity, and renal function were associated with worsening GFR during hospitalization, whereas immediate evidence-based treatment was unrelated to a change in GFR (Table 5).

**Table 5:** **Predictors of change in glomerular filtration rate from admission to discharge: multiple linear regression analysis results**

Independent variables	b	P value	CI 95%
Gender (M)	1.92	0.26	-1.418 to 5.262
Age (years)	-0.40	**<0.001**	-0.549 to -0.243
Immediate evidence based treatment	-0.84	0.74	-5.803 to 4.116
GFR at admission (mL/min/1.73 m^2^ )	-0.34	**<0.001**	-0.423 to 0.253
History of heart failure	-2.60	0.21	-6.704 to 1.513
History of PAD	4.04	0.16	-1.589 to 9.663
History of diabetes	1.00	0.55	-2.309 to 4.307
History of lipid disorders	-2.15	0.17	-5.212 to 0.915
History of hypertension	0.48	0.78	-2.908 to 3.862
Prior myocardial infarction	-0.36	0.83	-3.544 to 2.833
Prior stroke or TIA	-2.38	0.35	-7.436 to 2.675
Number of vessels with CAD	1.77	**0.03**	0.138 to 3.407
Ejection fraction (≤40)	-3.29	0.13	-7.497 to 0.924

Dialysis patients (n = 10) where excluded from the analysis.

Values in bold are statistically significant.

*Abbreviations:* *GFR* glomerular filtration rate, *CAD* coronary artery disease, *EF* ejection fraction, *PAD* peripheral arterial disease, *TIA* transient ischemic attack.

In-hospital mortality was low, with only six deaths (1.8%) total (Table 1). Patients who died during the hospitalization had variable admission renal function: their aGFR ranged from 20.9 to 79.6 mL/min/1.73 m2. Among the 317 patients with a positive link to the SISEPS register, the 1–year mortality rate was 15.3% and the 2-year mortality rate was 21.3%.

## Discussion

Our results indicate that 43.1% of patients hospitalized for NSTEMI had renal dysfunction at admission. This percentage is considerably higher than in the general population, confirming the relationship between CVD and renal dysfunction [7–10]. CAD was more severe in patients with more severe kidney damage, and the number of patients with three-vessel CAD increased with increasing renal dysfunction. Our study showed this relationship, which had previously been reported only for registry studies [11]. We also found that CRP increased with increasing renal dysfunction and that its levels were associated with the extent of CAD, suggesting that this proteinas inflammation mediator represents a link between renal dysfunction and CAD.

Recent research has focused on the role of inflammatory markers as a link between CVD and kidney disease. CKD is characterized by chronic inflammation, and inflammation also plays an integral role in accelerated atherosclerosis. Systemic microinflammation determines the extent of vascular endothelium damage, which is a key risk factor for both acute coronary syndrome (ACS) and CKD progression [12, 13]. Patients with ACS and increased CRP have a worse outcome than those with normal levels of inflammatory markers [14]. Moreover, CRP is an important predictor of ACS. Inflammatory activation may also play a role in heart failure by contributing to vascular dysfunction and fluid overload [15], thereby leading to inadequate renal perfusion pressure, peritubular edema, pathological reduction of glomerular filtration, and mixed inflammatory and ischemic tubular damage [16]. These effects may explain why heart failure on admission was more frequent in our patients with CKD and was directly related to the extent of renal damage. Our finding of an association between UA levels and renal dysfunction is consistent with previously reported associations between UA and obesity, hypertension, metabolic syndrome, and glucose intolerance, all of which contribute to the pathogenesis of CVD [17, 18]. Although some epidemiological, experimental, and clinical data have implicated UA in the pathogenesis of kidney injury [19], other studies have not demonstrated a clear effect of UA in the pathogenesis of endothelial damage [20]. Conversely, UA levels were unrelated to the number of diseased coronary arteries. These results suggest that increased UA may be the consequence of various factors, such as reduced urinary excretion due to renal dysfunction, cardiac disease, hypertension, or metabolic syndrome [21]. Alternately, they might be involved in the early phases of vascular damage [22].

Our observation that patients with renal dysfunction at admission were older than those without renal dysfunction is consistent with previous findings [23]. Furthermore, older patients had worsening of their GFR during hospitalization. Patients with severe admission renal dysfunction had a lower diastolic and mean arterial pressure, which concurs with a recent report that diastolic hypotension is more frequent in patients with stage 3 and 4 CKD [24]. These observations may be partially explained by age-related decreases in GFR, due to reduced arterial compliance, changes in afterload, and diastolic dysfunction [25, 26].

Moreover, excluding from the study population patients in chronic dialysis, patients with severe renal dysfunction at admission also presented a more substantial improvement in GFR at discharge: these data may reflect the effect on renal function of the patient’s clinical condition stabilization. Other events may also have contributed, such as the discontinuation of ACE inhibitors, but we are not able to verify this hypothesis because we did not analyze patients’ medications before hospital admission but during the first 24 hours. In these period, ACE inhibitors were administered to all patients in the absence of the contraindications (hyperkaliemia, hypotension), independently of the presence or absence of renal dysfunction.

Our data confirm the use of evidence-based therapy in a high percentage of patients with NSTEMI and a careful use of multiple drugs in patients with severe renal dysfunction. Interestingly, use of clopidogrel did not differ among the study groups, but patients with severe renal dysfunction were less likely to receive immediate evidence-based treatment in the first 24 hours after admission. Patients with renal dysfunction had a longer hospitalization, although this association became weaker after adjusting for gender, age, immediate treatment, and comorbidities. Our in-hospital mortality rate was low (1.8%), which may reflect the quality of care received and/or exclusion of patients with severe infection. Our small number of deaths prohibited determining whether mortality was associated with aGFR or immediate evidence-based treatment, as suggested by other authors [27–30]. Nevertheless, 1-year and 2-year mortality rates were 15.3% and 21.3%, confirming NSTEMI as a life-threatening condition.

The study is limited by its retrospective-observational nature and the relatively small sample size. Data regarding renal function before admission was frequently not available in the patients’ charts.

Thus, we cannot confirm that the aGFR values represent the patients’ baseline value. Nevertheless, because the time from NSTEMI onset to admission was minimal and creatinine is a late marker of renal function changes, aGFR probably at least reflected the renal function associated with the hemodynamic changes related to NSTEMI. Moreover, only a single CRP value was available for all patients.

## Conclusions

Our results indicate that, among patients hospitalized for NSTEMI, those with a severe degree of renal dysfunction had more diffuse CAD. CRP was also positively correlated with renal dysfunction, confirming its possible use as a biomarker of the extent of coronary atherosclerosis in patients with CKD. The role of UA is unclear, as there was no association between UA and the number of diseased coronary arteries.

## References

[1] Shani S, Sarnak MJ (2010). Cardiovascular disease and CKD: Core Curriculum 2010. Am J Kidney Dis.

[2] Libby P, Ridker PM, Hansson GK (2009). Inflammation in atherosclerosis: from pathophysiology to practice. J Am Coll Cardiol.

[3] Hansson KK (2005). Inflammation. Atherosclerosis and coronary artery disease. N Engl J Med.

[4] Anderson JL, Adams CD, Antman EM, Bridges CR, Callif RM, Casey DE, Chavey WE, Fesmire FM, Hochman JS, Levin TN, Lincoff AM, Peterson ED, Theroux P, Wenger NK, Wright RS (2012). ACC/AHA Focused update incorporated into the ACC/AHA 2007 guidelines for the management of patients with unstable Angina/Non-ST-elevation myocardial infarction: a report of the American college of cardiology foundation/American heart association task force on practice guidelines. Circulation.

[5] Hamm CW, Bassand JP, Agewall S, Bax J, Boersma E, Bueno H, Caso P, Dudek D, Gielen S, Huber K, Ohman M, Petrie MC, Sonntag F, Sousa Uva M, Storey RF, Wijns W, Zahger D (2011). ESC Guidelines for the management of acute coronary syndromes in patients presenting without persistent ST-segment elevation. The Task Force for the management of acute coronary syndromes (ACS) in patients presenting without persistent ST-segment elevation of the European Society of Cardiology (ESC). Eur Heart J.

[6] **The SIEMENS Dimension EXL TM integrated chemistry system** LOCI Module. Issue Date 2012–11–13. http://www.healthcare.siemens.com/integrated-chemistry/systems/dimension-exl-200-integrated-chemsys/assays

[7] Centers for Disease Control and Prevention (2010). National Chronic Kidney Disease Fact Sheet: General Information and National Estimates on Chronic Kidney Disease in the United States. 2010.

[8] Fox CS, Muntner P, Chen AY, Alexander KP, Roe MT, Cannon CP, Saucedo JF, Kontos MC, Wiviott SD (2010). Use of evidence –based therapies in short term out comes of ST-segment elevation myocardial infarction and non- ST-segment elevation myocardial infarction in patients with chronic kidney disease: A report from the national cardiovascular data acute coronary treatment and intervention outcomes network registry. Circulation.

[9] Han JH, Chandra A, Mulgund J, Roe MT, Peterson ED, Szczech LA, Patel U, Ohman EM, Lindsell CJ, Gibler WB (2006). Chronic kidney disease in patients with Non- STsegment elevation acute coronary syndromes. Am J Med.

[10] Rogers WJ, Canto JG, Lambrew CT, Tiefenbrunn AJ, Kinkaid B, Shoultz DA, Frederick PD, Every N (2000). Temporal trends in the treatment of over 1.5 million patients with myocardial infarction in the US from 1990 through 1999: the National Registry of Myocardial Infarction 1. 2 and 3. J Am Coll Cardiol.

[11] Hanna EB, Chen AY, Roe MT, Sausedo JF (2012). Characteristics and in-hospital outcomes of patients presenting with non ST-segment elevation myocardial infarction found to have significant coronary artery disease on coronary angiography and managed medically: stratification according to renal function. Am Heart J.

[12] Crea F, Liuzzo G (2013). Pathogenesis of acute coronary syndromes. J Am Coll Cardiol.

[13] Rosner MH, Ronco C, Okusa MD (2012). The role of inflammation in the cardio-renal syndrome: a focus on cytokines and inflammatory mediators. Semin Nephrol.

[14] Kaski JC (2010). C-reactive protein improves risk prediction in patients with acute coronary syndrome. or does it?. Eur Heart J.

[15] McCullough PA, Omland T, Maisel AS (2003). B-type natriuretic peptides: a diagnostic breakthrough for clinicians. Rev Cardiovasc Med.

[16] Ronco C, Cicoira M, McCullough PA (2012). Cardiorenal syndrome type 1 pathophysiological crosstalk leading to combined heart and kidney dysfunction in the setting of acutely decompensated heart failure. J Am Coll Cardiol.

[17] Milionis HJ, Kakafika AI, Tsouli SG, Athyros VG, Bairaktari ET, Seferiadis KI, Elisaf MS (2004). Effects of statin treatment on uric acid homeostasis in patients with primary hyperlipidemia. Am Heart J.

[18] Cappuccio FP, Strazzullo P, Farinaro E, Trevisan M (1993). Uric acid metabolism and tubular sodium handling. Results from a population-based study. JAMA.

[19] Ejaz AA, Mu W, Kang DH, Roncal C, Sautin YY, Henderson G, Tabah-Fisch I, Keller B, Beaver TM, Nakagawa T, Johnson RJ (2007). Could uric acid have a role in acute renal failure?. Clin J Am Soc Nephrol.

[20] Culleton BF, Larson MG, Kannel WB (1999). Serum uric acid and risk for cardiovascular disease and death: the Framingham heart study. Ann Intern Med.

[21] Feig DI, Kang DH, Johnson RJ (2008). Uric acid and cardiovascular disease. N Engl J Med.

[22] Mazzali M, Hughes J, Kim YG (2001). Elevated uric acid increases blood pressure in the rat by a novel crystal-indipendent mechanism. Hypertension.

[23] Santopinto JJ, Fox KA, Goldberg RJ, Budaj A, Piñero G, Avezum A, Gulba D, Esteban J, Gore JM, Johnson J, Gurfinkel EP, GRACE Investigators (2003). Creatinine clearance and adverse hospital outcomes in patients with acute coronary syndromes: findings from the global registry of acute coronary events (GRACE). Heart.

[24] Tomlinson LA, Holt SG, Leslie AR, Rajkumar C (2009). Prevalence of ambulatory hypotension in elderly patients with CKD stage 3 and 4. Nephrol Dial Transplant.

[25] Lakatta E, Gerstenblith G, Weisfeldt M, Braunwald E (1997). The Aging Heart: Structure. Function and Disease. Heart Disease.

[26] Douville P, Martel A, Talbot J, Desmeules S, Langlois S, Agharazii M (2009). Impact of age on glomerular filtration estimates. Nephrol Dial Transplant.

[27] Gibson CM, Dumaine RL, Gelfand EV, Murphy SA, Morrow DA, Wiviott SD, Giugliano RP, Cannon CP, Antman EM, Braunwald E, TIMI Study Group (2004). Association of glomerular filtration rate on presentation with subsequent mortality in non-ST-segment elevation acute coronary syndrome; observations in 13,307 patients in five TIMI trials. Eur Heart J.

[28] Mueller C, Neumann FJ, Perruchoud AP, Buettner HJ (2004). Renal function and long term mortality after unstable angina/non-ST segment elevation myocardial infarction treated very early and predominantly with percutaneous coronary intervention. Heart.

[29] Hanna EB, Chen AY, Roe MT (2011). Characteristics and in-hospital outcomes of patients with non-ST-segment elevation myocardial infarction and chronic kidney disease undergoing percutaneous coronary intervention. JACC Cardiovasc Interv.

[30] Khambatta S, Farkouh ME, Wright RS, Reeder GS, McCullough PA, Smars PA, Hickson LJ, Best PJ (2012). Chronic kidney disease as a risk factor for acute coronary syndromes in patients presenting to the emergency room with chest pain. Transl Res.

[CR31] The pre-publication history for this paper can be accessed here:http://www.biomedcentral.com/1471-2369/15/152/prepub

